# The health status, social support, and subjective well-being of older individuals: evidence from the Chinese General Social Survey

**DOI:** 10.3389/fpubh.2024.1312841

**Published:** 2024-01-25

**Authors:** Yuchen Zhang, Longyu Sun

**Affiliations:** ^1^Department of Sociology, Sungkyunkwan University, Seoul, Republic of Korea; ^2^School of Foreign Languages, Shandong Jianzhu University, Jinan, China

**Keywords:** mental health, physical health, social support, subjective well-being, mediating analysis

## Abstract

**Objectives:**

This study aims to investigate the impact of health status and social support on the subjective well-being of older individuals.

**Methods:**

Using data from the China General Social Survey 2017, this research analyzed 5,187 Chinese citizens aged 60 years and older. The predicted effect of each variable on subjective well-being was evaluated through hierarchical regression analysis. The direct and indirect effects of social support and health status on subjective well-being are examined based on a structural equation model.

**Results:**

The mental health and social support positively impact subjective well-being. Mental health mediates the effect of physical health on subjective well-being, and social support mediates the relationship between physical and mental health and subjective well-being.

**Conclusion:**

The findings provide strong evidence for the interrelationship mechanisms among the factors influencing subjective well-being. Consequently, improving mental health services and social support systems is advantageous for enhancing the well-being of Chinese seniors.

## 1 Introduction

The global population is undergoing unprecedented age structure changes marked by extended life expectancy, rapid population growth, and a rising proportion of seniors (1). China, entering the aging society in 2000, now boasts the world’s largest older population, surpassing 264 million (2). Anticipated to exceed 400 million by 2035, constituting over 30% of the population, China faces deepening aging issues (3). Irreversible aging trends have spurred interest in active aging, focusing on the subjective well-being of older individuals as a vital measure for successful aging and a critical societal goal (4, 5).

Subjective well-being (SWB) is a comprehensive assessment of living quality based on personal expectations, marked by subjectivity, stability, and wholeness (6). It has become a key policy goal in many nations, with some Chinese regional governments using citizens’ SWB to evaluate officials (7, 8). SWB is recognized as integral to successful aging (9); studies suggest a U-shaped trajectory of well-being, rising with age before declining, notably after 70 years (10, 11). SWB, influenced by economic, social, and health factors, undergoes continuous changes across one’s lifespan (12, 13).

China’s rapid aging is coupled with concerning health issues among older individuals (14), with up to 75% facing chronic conditions (15). Diminished well-being is associated with various diseases (16). Mental health is as crucial as physical health in older adults (17, 18). Research shows that long-term physical health problems often lead to mental health problems like depression and anxiety, reducing subjective well-being and quality of life (19, 20). The close connection between health and SWB grows with age (21, 22). Additionally, as older adults’ health deteriorates and their social roles change, they struggle to adapt to these dramatic changes to maintain average well-being, and it is necessary to have social support to cope with these changes and the gradual loss of resources (23, 24). Social support, acting as an external resource through emotional support, substantive support, and information support avenues, mitigates life stress for older individuals, promoting positive emotional experiences and helping to maintain or improve the quality of life (25). Among the various social determinants, social support emerges as a substantiated protective factor for SWB, potentially mediating the link between older people’s health status and SWB (26).

Health and social support are prerequisites for a person to have well-being, and the progression of active aging is inextricably linked to the well-being of older people (27–30). Older individuals are an essential group in society, and their health and well-being are intimately related to the stability and advancement of society (31). By examining the role of social support as an intermediary, we can gain a more comprehensive awareness of the relevant factors that affect SWB in older individuals, which is of considerable practical value for promoting the development and optimization of the system of old-age services. Therefore, this study aims to consider older people’s unique mental and physical characteristics, uncover the mechanisms of health status and social support on SWB, and provide a scientific foundation for enhancing their living quality, formulating social policy, and helping them overcome challenges in later life.

**Figure 1:**
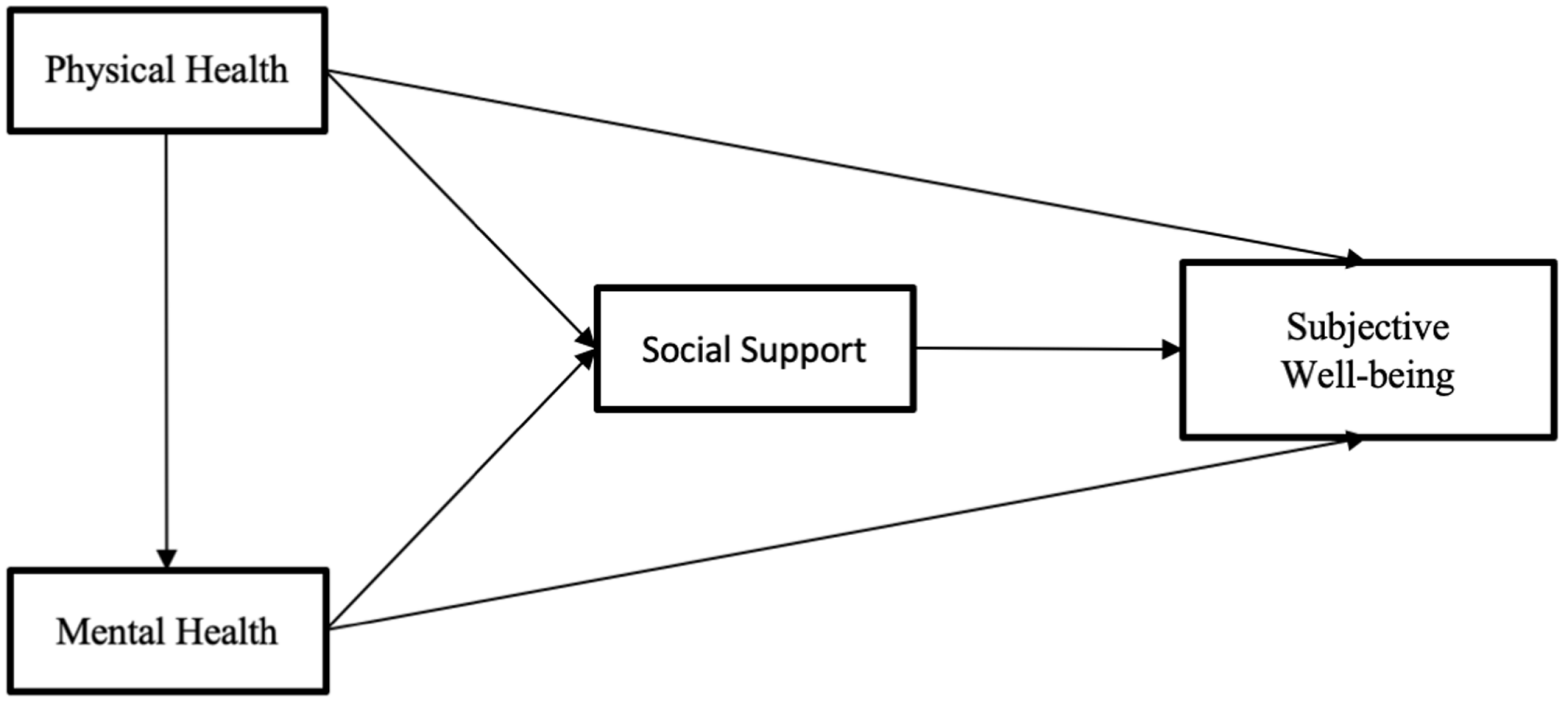
A hypothetical model: direct and indirect pathways between study variables.

We propose the following hypotheses regarding the relationship between older individuals’ health status, social support, and SWB based on the available evidence, Figure 1:

*H1*: Physical health positively impacts the SWB of older individuals.

*H2*: Mental health positively impacts the SWB of older individuals.

*H3*: Mental health mediates the relationship between physical health and SWB.

*H4*: Social support positively impacts the SWB of older individuals.

*H5*: Social support mediates the relationship between mental health, physical health, and SWB.

## 2 Research methods

### 2.1 Data sources

This study uses data from CGSS 2017 to conduct an empirical study. The CGSS is China’s first nationwide, ongoing, comprehensive professional survey program (32). It used a multi-stage stratified probability sampling method to conduct a household survey in 28 provinces, municipalities, and autonomous regions of China, with questions at the social, community, household, and individual levels, and a total of 12,582 data items were collected. The CGSS 2017 stands out for its comprehensive coverage and representativeness; the questionnaire demonstrates high levels of authenticity and reliability (33). The subject of this study was older people aged 60 years and older, so the sample data of respondents below 60 years were excluded. After selecting the required variables in the database according to the research design, invalid samples such as “refused to answer,” “not applicable,” and “do not know” were removed. Finally, we obtained 5,187 valid samples.

### 2.2 Measures

#### 2.2.1 Health status

This study evaluated the health status of older people from two different perspectives: mental and physical. The physical health of older people is a visual representation of their health status; therefore, question A15, “How do you feel about your current physical health?” was chosen as the primary variable. Mental health is a comprehensive assessment of the mental condition of older people, and question A17, “In the past 4 weeks, how often have you felt depressed or upset?” was chosen as the primary variable of this research. The responses to both questions were rated on a scale from 1 to 5, with higher scores indicating a healthier condition.

#### 2.2.2 Social support

The concept of social support encompasses many aspects, and most social support metrics are also examined across multiple dimensions. According to Wei et al. (34), social support comprises three components: social class identity, social interaction, and social security, are given as follows:

**Table:** 

Variables	Measurement questions	Definition
Social Class Identity	A43a: In general, at what level do you currently stand in society?	1–10 points, the more significant the score, the greater the class identity of the individual.
	A43b: At what level, do you think you were 10 years ago?	
	A43c: What level do you think you will be at in 10 years?	
Social Interaction	A31 (1): In the past year, did you frequently participate in social activities during your leisure time?	The higher the frequency, the greater the score and the more social interaction.
	A30 (6): Did you frequently get together with non-living relatives in leisure time during the past year?	
	A31a: How frequently do you participate in social recreation with your neighbors?	
	A31b: How frequently do you participate in social or recreational activities with other friends?	
Social Security	A61: Are you enrolled in the following social security programs? (basic urban medical insurance/new rural cooperative medical insurance/free medical service, basic urban/rural pension insurance, commercial medical insurance, and commercial pension insurance)	Yes is 1 point, otherwise 0. The greater the number of projects participants participate in, the higher the social security.

#### 2.2.3 Subjective well-being

Subjective well-being is a person’s assessment of their living quality following self-defined standards and is classified into different levels of subjective perception. In CGSS 2017, question A36, “Overall, do you feel that you are happy in your life?” was selected and rated on a scale from one to five, with a more significant score meaning greater subjective well-being.

#### 2.2.4 Basic variables

Individual demographic variables and socioeconomic factors also affect the SWB of older individuals, which were included as controlled variables to decrease the interference of individual characteristics of the study subjects and external environmental factors on the study results, including the following:

**Table:** 

Variable	Definition
Sex	Male = 1, Female = 0
Age	60–69 years = 1, 70–79 years = 2, 80–89 years = 3, and Above 90 years = 4
Education	Uneducated = 1, Primary school = 2, Middle school = 3, High school or technical secondary school = 4, and College and above = 5
Political affiliation	Communist Party = 1, Non-communist party = 0
Household type	Rural = 1, Urban = 0
Marital status	With marriage = 1, No marriage = 0
Economic status	Below average level = 1, Average level = 2, Above average level = 3

### 2.3 Statistical analysis

The SPSS 26.0 and RStudio statistical programs were used for the data analysis. A statistical analysis of demographic variables was conducted employing descriptive statistics. The demographic differences in older individuals’ subjective well-being were compared using the independent sample T-test and single-factor ANOVA. The correlation between the health status, social support, and SWB of older individuals was determined using Pearson correlation analysis. Exploring the factors influencing the SWB of older individuals using linear regression analysis. In addition, a structural equation model (SEM) is established to assess the proposed hypothesis model’s fit with the data (35, 36). The model was examined by maximum likelihood estimation. The model’s fit was judged using the chi-square statistic (χ^2^/df ≤ 5), the Tucker-Lewis index (TLI ≥ 0.90), the comparative fit index (CFI ≥ 0.90), the root mean square error of approximation (RMSEA≤0.05), and the standardized root mean square residual (SRMR≤0.05) (37, 38). Testing the mediation effect of the model with the Bootstrap test, the significance of the indirect effects was confirmed using the 95% bias-corrected confidence intervals from 5,000 Bootstrap samples (39, 40). If “0″ was not contained within the confidence interval, the indirect effect is regarded as statistically significant, and when *p* < 0.05, statistical significance was assumed to exist (41).

## 3 Results

### 3.1 Characteristics of demographic variables and differences in SWB

This study included 5,187 older individuals, 2,500 (48.2%) male and 2,687 (51.8%) female. The age range was between 60 and 109 years, with an average age of 71.88 and a standard deviation of 8.48. Among respondent groups, 49.8% had primary school education or less, 85.9% were non-communist party members, 52.4% were in rural households, 75.9% were married, and 93.2% of older people were at an average or below economic level. In terms of sex, age, education level, political affiliation, household type, marital status, and economic status, the differences in SWB of older individuals were statistically significant (*p* < 0.05), as shown in Table 1.

**Table 1:** Descriptive statistics of demographic variables and differences in subjective well-being scores (*N* = 5,187).

Variable	*n* (%)	M ± SD	*t/F*	*p*
Sex			−2.252	0.024
Male	2,500 (48.2)	3.85 ± 0.86		
Female	2,687 (51.8)	3.90 ± 0.88		
Age (years)		71.88 ± 8.48	14.898	<0.001
60–69	2,297 (44.3)	3.79 ± 0.90		
70–79	1,887 (36.4)	3.91 ± 0.83		
80–89	834 (16.1)	4.00 ± 0.86		
Above 90	169 (3.3)	3.95 ± 0.86		
Education level			18.157	<0.001
Uneducated	1,125 (21.7)	3.71 ± 0.99		
Primary school	1,458 (28.1)	3.86 ± 0.87		
Middle school	1,387 (26.7)	3.93 ± 0.83		
High school or technical secondary school	845 (16.3)	3.95 ± 0.82		
College and above	372 (7.2)	4.07 ± 0.72		
Political affiliation			8.993	<0.001
Communist Party	732 (14.1)	4.10 ± 0.69		
Non-communist party	4,455 (85.9)	3.84 ± 0.90		
Household type			9.147	<0.001
Rural	2,716 (52.4)	3.77 ± 0.92		
Urban	2,471 (47.6)	3.99 ± 0.81		
Marital status			4.669	<0.001
With marriage	3,935 (75.9)	3.91 ± 0.85		
No marriage	1,252 (24.1)	3.77 ± 0.95		
Economic status			249.317	<0.001
Below average level	2,550 (49.2)	3.62 ± 0.96		
Average level	2,283 (44.0)	4.09 ± 0.69		
Above average level	354 (6.8)	4.35 ± 0.70		

### 3.2 Scores and correlations between variables

Table 2 presents the findings of a correlation analysis performed on the research variables. As seen from the results, mental health was discovered to have a positive connection with physical health; social support was positively associated with both mental and physical health; and mental health, physical health, and social support were positively correlated with SWB (*p* < 0.01).

**Table 2:** Health status, social support, and subjective well-being scores and correlations in older individuals (*N* = 5,187).

Variables	M ± SD	1	2	3	4
1. Physical health	3.07 ± 1.09	1			
2. Mental health	3.74 ± 1.03	0.428^**^	1		
3. Social support	26.42 ± 7.22	0.249^**^	0.199^**^	1	
4. Subjective well-being	3.87 ± 0.87	0.248^**^	0.343^**^	0.286^**^	1

^**^p < 0.01.

### 3.3 Hierarchical regression analysis

Subjective well-being served as the dependent variable in the model. Simultaneously, physical health, mental health, and social support have been included as independent variables; demographic variables that were statistically significant after univariate analysis were used as control variables in the regression equation, and hierarchical regression was conducted. Model 1 only included demographic control variables; all variables significantly affected SWB except for household type. Model 2 added health status variables based on Model 1, where physical health, mental health, sex, age, marital status, and economic status significantly affected SWB, explaining 19.2% of the variance. Model 3 added the variable of social support based on Model 2, where mental health, physical health, social support, sex, age, marital status, and economic status were influential factors in the SWB of older individuals, explaining 20.9% of the variance, as shown in Table 3.

**Table 3:** Hierarchical regression analysis of older individuals’ subjective well-being (*N* = 5,187).

Variables	Model 1	Model 2	Model 3
	*β*(SE)	*β*(SE)	*β*(SE)
Physical health		0.110(0.011)^**^	0.088(0.011) ^**^
Mental health		0.252(0.012)^**^	0.244(0.012) ^**^
Social support			0.147(0.002) ^**^
Sex	−0.060(0.024)^**^	−0.077(0.023)^**^	−0.067(0.023) ^**^
Age (years)	0.105(0.015)^**^	0.114(0.015)^**^	0.113(0.014) ^**^
Education level	0.047(0.012)^**^	0.011(0.012)	0.006(0.012)
Political status	0.030(0.036)^*^	0.022(0.035)	0.018(0.034)
Household type	0.027(0.028)	−0.016(0.026)	−0.015(0.026)
Marital status	0.079(0.029)^**^	0.064(0.027)^**^	0.064(0.027) ^**^
Economic status	0.261(0.020)^**^	0.204(0.019)^**^	0.151(0.020) ^**^
*R* ^2^	0.105	0.192	0.209
Adjusted *R*^2^	0.103	0.190	0.207
*F*	86.401^**^	136.278^**^	136.587^**^

^*^p < 0.05, ^**^p < 0.01. Dependent variables: subjective well-being. Independent variable: Model 1: demographic control variables; Model 2: demographic control variables and health status variables; Model 3: demographic control variables, health status variables, and social support.

### 3.4 Structural equation model analyses

**Figure 2:**
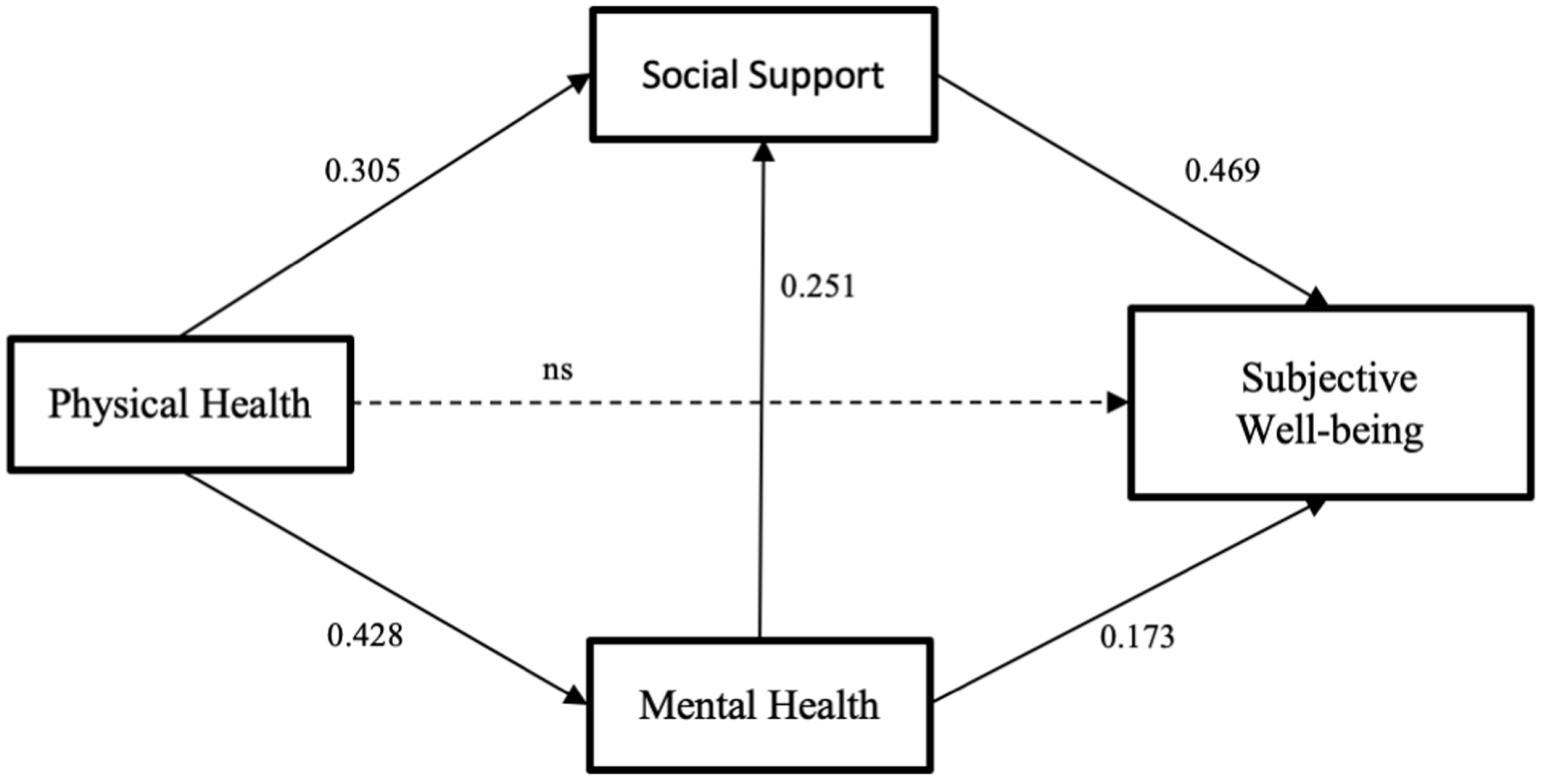
The multiple indirect effects model with standardized coefficients.

The hypothetical model is tested using SEM and path analysis to assess the model’s mediation effect. The fit of the structural model was calculated to determine whether the structural model aligns with the theoretical model. Table 4 displays the model’s fit indices, and all indicators have appropriate values, indicating that this model achieves a satisfactory fit. Figure 2 shows the specific structural model.

**Table 4:** Fit indices for structural equation models (*N* = 5,187).

	χ^2^	df	CFI	TLI	RMSEA(90%CI)	SRMR
Model	22.299	6	0.994	0.985	0.023[0.013–0.033]	0.011

Next, the bootstrap method (5,000 draws) was used to assess the significance of the multiple indirect effects in the study model. The indirect effects and 95% confidence intervals are shown in Table 5. Mental health and social support both have a significant direct effect on SWB (*β* = 0.173; *β* = 0.469, *p* < 0.001), and mental health has a significant indirect effect on SWB via social support, indicating a partial mediation effect of social support in the association between mental health and SWB (*β* = 0.118, 95% CI = 0.075–0.136, *p* < 0.001). The direct effect of physical health on SWB was insignificant (*p* = 0.457). However, through mental health and social support, physical health exerts a significant indirect effect on SWB. Thus, mental health and social support entirely mediate the association between physical health and SWB, respectively (*β* = 0.074, 95% CI = 0.043–0.075; *β* = 0.143, 95% CI = 0.085–0.155, *p* < 0.001). Furthermore, the effect of physical health on SWB through mental health followed by social support was significant (*β* = 0.050, 95% CI = 0.030–0.055, *p* < 0.001).

**Table 5:** SEM standardized path coefficient estimation and bootstrap confidence intervals.

Model pathways	Estimated	95% Bias-corrected CI
MH″SS″SWB	0.118	0.075–0.136
PH″SS″SWB	0.143	0.085–0.155
PH″MH″SWB	0.074	0.043–0.075
PH″MH″SS″SWB	0.050	0.030–0.055

PH, Physical health; MH, Mental health; SS, Social support; and SWB, Subjective well-being.

## 4 Discussion

This study contributes to understanding the quality of life of older people in China by testing a comprehensive model of the effects of health status and social support on SWB. The model discusses the crucial roles of mental health, physical health, and social support on older people’s SWB. The analysis explores the direct and indirect relationships of these variables to uncover underlying mechanisms.

The findings suggest that physical health is an essential factor in the SWB of older individuals, but the direct effect on SWB is not significant, rejecting hypothesis H1. Mental health positively affects older individuals’ SWB, supporting hypothesis H2, which aligns with findings from prior studies (42, 43). Older adults with better mental health can have optimistic attitudes that enable them to cope effectively with life’s adversities and challenges, resulting in higher well-being. Mental health helps to enhance the coping capacity and adaptability of older people to confront problems, thus increasing their subjective well-being (44, 45). The older adults’ level of subjective well-being can be positively predicted by their mental health (46). Consistent with hypothesis H3, mental health mediates the association between physical health and SWB of older individuals, which implies that physical health can indirectly influence older individuals’ subjective well-being through mental health. A deterioration in an older person’s physical health can sometimes be followed by a fall in their mental health, and that leads to lower well-being. This is consistent with other literature that physical health can substantially impact the mental health of older individuals, and disability, chronic disease, insomnia, and other physical functions decreases in physical function can contribute to low self-esteem, anxiety, depression, and other mental issues in older individuals; physical frailty can quadruple the probability of depression (47, 48); patients in their later years who suffer ailments such as chronic lung disease, arthritis, and coronary heart disease have been shown to have elevated levels of low mood as well as diminished hedonic and eudemonic well-being (21). Results of our study reveal that mental health is positively associated with physical health and that older adults experience depression, frustration, or restlessness due to physical pain and impairment (49). Thus, physical health can influence mental health, which, in turn, can impact subjective well-being. By fostering an overall improvement in the mental health of older individuals, negative emotions due to physical health problems can be reduced, thereby improving subjective well-being (50, 51). The findings emphasize the importance of mental health among older adults, a finding that may have multiple implications at the individual, societal, and policy levels. For older adults, recognizing the positive association between mental health and SWB may motivate individuals to pay more attention to and maintain their mental health, such as by seeking mental health services and engaging in social activities, thereby improving their SWB. This result may enhance social awareness and attention to mental health problems, especially among the older population. Senior care organizations and social services are encouraged to consider the mental health needs of older people more comprehensively and provide more mental health support services, including social and recreational activities and mental health counseling, to improve the well-being of older people. Additionally, this may lead governments to focus more on the mental health of older people in their policy development, including increased investment in mental health services, the provision of more mental health resources, and the adoption of more comprehensive policies on the care of older people in order to enhance their overall quality of life and sense of well-being.

Social support has an immediate impact on the SWB of older individuals, supporting hypothesis H4, which is consistent with past research indicating that social support has a beneficial influence on older individuals’ SWB (52). That is, the more social support an individual perceives, utilizes, and receives, the higher well-being one can attain in senior living. Therefore, high levels of social support play a crucial role in enhancing the well-being of older individuals. Consistent with hypothesis H5, our findings indicate that social support mediates the association between older individuals’ physical and mental health and their SWB, suggesting that physical and mental health indirectly influence SWB through social support. Older individuals with better physical and mental health have a stronger self-identification, a greater probability of having positive relationships with others, can use available resources more positively, obtain higher social support, and experience greater personal well-being. This result concurs with findings of other research displaying that older individuals with better physical health can participate in more social activities, thereby increasing their identification with their social status, expanding their social circle, and gaining a tremendous amount of social support, which in turn contributes to and enhances their perception of well-being (53, 54); older individuals with better mental health tend to have a more optimistic mentality, and this mindset helps individuals feel more respect, support, and understanding in their social interactions, resulting in higher levels of social support, which expands their personal and social resources and raises their level of well-being (55); older people in better health feel more support from their family, friends, and society, and more significant quantities of available social support increase SWB in their later lives (56, 57). In addition, the model’s mediating effect validates the value-added spiral effect of the theory of resource conservation (58). That is, older individuals with high personal resources (physical health and mental health) are more inclined to develop new resources (social support), and the accumulation of such resources will lead to critical potential benefits (increased SWB). Compared with previous studies, the present study integrates physical and mental health factors and explains the mechanisms influencing SWB in older adults more comprehensively by considering social support as one of the critical factors connecting health status and SWB. The results emphasize the importance of social support for older adults, and focusing on social support while maintaining their physical and mental health may be an essential strategy for promoting overall well-being, which plays a vital role in establishing social identity, promoting interpersonal relationships, and providing psychological security. In addition, the results emphasize the need to strengthen the social support system, prompting social organizations and government agencies to pay more attention to providing and improving social support to better meet the spiritual needs of older people and improve their SWB.

However, this study has several limitations that need further improvement. Firstly, because the current study used a cross-sectional analysis, it could not determine the causal inference among health status, social support, and SWB. Future research should consider longitudinal designs to better understand the direction and causal relationships among variables. Secondly, in addition to health status and social support, additional variables, such as the way of old-age care and attitudes toward aging, affect the SWB of older individuals and need to be explored in future research. Thirdly, all of the variables measured in this research were those the participants self-reported, so it was impossible to exclude the influence of other factors, such as emotions and life stress, on the results. Fourthly, although the model in this study demonstrated a good fit, alternative models were not tested, so the possibility that other models could also fit adequately cannot be ruled out. Finally, this study is based on Chinese older adults. Due to cultural differences, regional differences, and national policy differences, the findings of this study may not apply to all older populations, especially those in Western countries. They could be further expanded to investigate other countries or ethnicities.

## 5 Conclusion

To summarize, this study used data from the CGSS 2017 and employed hierarchical regression analysis and SEM to analyze the influencing mechanisms of older individuals’ SWB from personal and social perspectives. The findings underscore the crucial roles of social support and mental health in promoting SWB among older people. To enhance the well-being of older individuals, the focus should be on improving social support through continuous enhancements to social security and medical insurance systems. Advocacy for active participation in society, fostering interpersonal relationships, and facilitating group support can solve problems like lack of mental comfort, insufficient financial support, and life care. Additionally, prioritizing mental health services for older individuals is vital. Governments and societies should provide targeted interventions, care, and positive psychological support for those at higher mental health risks and develop community-based social activities to further enrich their lives and improve mental health.

## Data availability statement

The datasets presented in this study can be found in online repositories. The names of the repository/repositories and accession number(s) can be found at: http://cgss.ruc.edu.cn/.

## Author contributions

YZ: Conceptualization, Data curation, Methodology, Software, Supervision, Writing – original draft. LS: Supervision, Validation, Writing – review & editing.

## References

[1] World Health Organization (2020). Decade of healthy ageing: Baseline report.

[2] Xu X Zhao Y Zhang X Xia S. Identifying the impacts of social, economic, and environmental factors on population aging in the Yangtze River Delta using the geographical detector technique. Sustain For. (2018) 10:1528. doi: 10.3390/su10051528

[3] Chen X Giles J Yao Y Yip W Meng Q Berkman L. The path to healthy ageing in China: a Peking University–lancet commission. Lancet. (2022) 400:1967–2006. doi: 10.1016/S0140-6736(22)01546-X, PMID: 36423650 PMC9801271

[4] Patel R Marbaniang SP Srivastava S Kumar P Chauhan S Simon DJ. Gender differential in low psychological health and low subjective well-being among older adults in India: with special focus on childless older adults. PLoS One. (2021) 16:e0247943. doi: 10.1371/journal.pone.0247943, PMID: 33684164 PMC7939372

[5] Stiglitz J. E. Sen A. Fitoussi J. P. (2009). Report by the commission on the measurement of economic performance and social progress. The Commission Paris.

[6] Chaurasia H Brajesh Sarode S. Exploring potential linkages between social support, retirement and subjective wellbeing among older Indians: does it a challenge to policy makers? Ageing Int. (2018) 43:207–36. doi: 10.1007/s12126-017-9317-3

[7] Bai C Gong Y Feng C. Social trust, pattern of difference, and subjective well-being. SAGE Open. (2019) 9:215824401986576. doi: 10.1177/2158244019865765

[8] Li L Zhang Z Fu C. The subjective well-being effect of public goods provided by village collectives: evidence from China. PLoS One. (2020) 15:1–16. doi: 10.1371/journal.pone.0230065 PMC7065793 32160249

[9] Lukaschek K Vanajan A Johar H Weiland N Ladwig K-H. “In the mood for ageing”: determinants of subjective well-being in older men and women of the population-based KORA-age study. BMC Geriatr. (2017) 17:1–9. doi: 10.1186/s12877-017-0513-5 28622764 PMC5474017

[10] Baird BM Lucas RE Donnellan MB. Life satisfaction across the lifespan: findings from two nationally representative panel studies. Soc Indic Res. (2010) 99:183–203. doi: 10.1007/s11205-010-9584-9, PMID: 21113322 PMC2990956

[11] Carstensen LL Turan B Scheibe S Ram N Ersner-Hershfield H Samanez-Larkin GR. Emotional experience improves with age: evidence based on over 10 years of experience sampling. Psychol Aging. (2011) 26:21–33. doi: 10.1037/a0021285, PMID: 20973600 PMC3332527

[12] Cheng G He S He Q Xie X Tian G Jiang N. Gender and residence differences in the association between social support and subjective well-being among Chinese oldest-old: a national longitudinal study. Arch Gerontol Geriatr. (2022) 98:104545. doi: 10.1016/j.archger.2021.104545, PMID: 34700136

[13] Steptoe A. Happiness and health. Annu Rev Public Health. (2019) 40:339–59. doi: 10.1146/annurev-publhealth-040218-044150 30601719

[14] Zhang YL Wu BJ Chen P Guo Y. The self-rated health status and key influencing factors in middle-aged and elderly: evidence from the CHARLS. Medicine. (2021) 100:e27772. doi: 10.1097/MD.0000000000027772, PMID: 34797304 PMC8601322

[15] Zhang R Lu Y Shi L Zhang S Chang F. Prevalence and patterns of multimorbidity among the elderly in China: a cross-sectional study using national survey data. BMJ Open. (2019) 9:e024268. doi: 10.1136/bmjopen-2018-024268, PMID: 31427309 PMC6701688

[16] Enkvist Å Ekström H Elmståhl S. What factors affect life satisfaction (LS) among the oldest-old? Arch Gerontol Geriatr. (2012) 54:140–5. doi: 10.1016/j.archger.2011.03.013, PMID: 21555158

[17] Henning G Bjälkebring P Stenling A Thorvaldsson V Johansson B Lindwall M. Changes in within- and between-person associations between basic psychological need satisfaction and well-being after retirement. J Res Pers. (2019) 79:151–60. doi: 10.1016/j.jrp.2019.03.008

[18] Stenling A Henning G Bjälkebring P Tafvelin S Kivi M Johansson B. Basic psychological need satisfaction across the retirement transition: changes and longitudinal associations with depressive symptoms. Motiv Emot. (2021) 45:75–90. doi: 10.1007/s11031-020-09854-2

[19] Malone C Wachholtz A. The relationship of anxiety and depression to subjective well-being in a mainland Chinese sample. J Relig Health. (2018) 57:266–78. doi: 10.1007/s10943-017-0447-4, PMID: 28702737 PMC5764815

[20] Soósová MS Timková V Dimunová L Mauer B. Spirituality as a mediator between depressive symptoms and subjective well-being in older adults. Clin Nurs Res. (2021) 30:707–17. doi: 10.1177/1054773821991152 33514280

[21] Steptoe A Deaton A Stone AA. Subjective wellbeing, health, and ageing. Lancet. (2015) 385:640–8. doi: 10.1016/S0140-6736(13)61489-0, PMID: 25468152 PMC4339610

[22] Steptoe A Leigh ES Kumari M. Positive affect and distressed affect over the day in older people. Psychol Aging. (2011) 26:956–65. doi: 10.1037/a0023303, PMID: 21517182

[23] Ngamaba KH Panagioti M Armitage CJ. How strongly related are health status and subjective well-being? Systematic review and meta-analysis. Eur J Pub Health. (2017) 27:879–85. doi: 10.1093/eurpub/ckx081, PMID: 28957478

[24] Wang X. Subjective well-being associated with size of social network and social support of elderly. J Health Psychol. (2016) 21:1037–42. doi: 10.1177/1359105314544136, PMID: 25104778

[25] Liu N He Y Li Z. The relationship between internet use and self-rated health among older adults in China: the mediating role of social support. Int J Environ Res Public Health. (2022) 19:14785. doi: 10.3390/ijerph192214785, PMID: 36429504 PMC9690403

[26] Mo PK Wong EL Yeung NC Wong SY Chung RY Tong AC. Differential associations among social support, health promoting behaviors, health-related quality of life and subjective well-being in older and younger persons: a structural equation modelling approach. Health Qual Life Outcomes. (2022) 20:38. doi: 10.1186/s12955-022-01931-z, PMID: 35246166 PMC8895671

[27] Dong X Bergren S Wang B Kozlov E. The associations between social support and negative social interaction with suicidal ideation in US Chinese older adults. Aging Ment Health. (2021) 25:94–8. doi: 10.1080/13607863.2019.1680953, PMID: 31650846

[28] Jarosz E. Lifestyle differentiation among older adults: exploring the links between individuals’ behaviours, socio-demographic characteristics, health and wellbeing in later life. Ageing Soc. (2021) 43:2157–72. doi: 10.1017/S0144686X21001586

[29] Li B Ma H Guo Y Fuming X Yu F Zhou Z. Positive psychological capital: a new approach to social support and subjective well-being. Soc Behav Personal Int J. (2014) 42:135–44. doi: 10.2224/sbp.2014.42.1.135

[30] Veenstra M Daatland SO Aartsen M. The role of subjective age in sustaining wellbeing and health in the second half of life. Ageing Soc. (2021) 41:2446–66. doi: 10.1017/S0144686X2000032X

[31] Wiesmann U Hannich H-J. A salutogenic view on subjective well-being in active elderly persons. Aging Ment Health. (2008) 12:56–65. doi: 10.1080/13607860701365998, PMID: 18297479

[32] China CGSS. Chinese general social survey: Digital chronicle of Chinese social change. (2023). Available at: http://cgss.ruc.edu.cn/English/Home.htm (Accessed December 8, 2023).

[33] Liu P Cao J Nie W Wang X Tian Y Ma C. The influence of internet usage frequency on Women’s fertility intentions—the mediating effects of gender role attitudes. Int J Environ Res Public Health. (2021) 18:4784. doi: 10.3390/ijerph18094784, PMID: 33946141 PMC8124929

[34] Wei Q Su HY Lv J Jin CY. Northwest Popul J. (2020) 41:106–15. doi: 10.15884/j.cnki.issn.1007-0672.2020.05.009

[35] Ullman JB Bentler PM. Structural equation modeling. In: Irving BW, editor. Handbook of psychology. Vol. 2. 2nd ed. John Wiley & Sons, Inc. (2012). p. 661–690.

[36] West SG Taylor AB Wu W. Model fit and model selection in structural equation modeling. In: Hoyle RH, editor. Handbook of structural equation modeling. New York: The Guilford Press (2012). p. 209–31.

[37] Maydeu-Olivares A. Maximum likelihood estimation of structural equation models for continuous data: standard errors and goodness of fit. Struct Equ Model Multidiscip J. (2017) 24:383–94. doi: 10.1080/10705511.2016.1269606

[38] Schermelleh-Engel K Moosbrugger H Müller H. Evaluating the fit of structural equation models: tests of significance and descriptive goodness-of-fit measures. Methods Psychol Res Online. (2003) 8:23–74.

[39] Banjanovic ES Osborne JW. Confidence intervals for effect sizes: applying bootstrap resampling. Pract Assess Res Eval. (2016) 21:5. doi: 10.7275/DZ3R-8N08

[40] Preacher KJ Hayes AF. Asymptotic and resampling strategies for assessing and comparing indirect effects in multiple mediator models. Behav Res Methods. (2008) 40:879–91. doi: 10.3758/BRM.40.3.879, PMID: 18697684

[41] MacKinnon D. Introduction to Statistical Mediation Analysis. New York: Routledge. (2012).

[42] Kohn JN Jester DJ Dilmore AH Thomas ML Daly R Jeste DV. Trends, heterogeneity, and correlates of mental health and psychosocial well-being in later-life: study of 590 community-dwelling adults aged 40–104 years. Aging Ment Health. (2023) 27:1198–207. doi: 10.1080/13607863.2022.2078790, PMID: 35622016

[43] Lombardo P Jones W Wang L Shen X Goldner EM. The fundamental association between mental health and life satisfaction: results from successive waves of a Canadian national survey. BMC Public Health. (2018) 18:342. doi: 10.1186/s12889-018-5235-x, PMID: 29530010 PMC5848433

[44] Fuller HR Huseth-Zosel A. Lessons in resilience: initial coping among older adults during the COVID-19 pandemic. The Gerontologist. (2021) 61:114–25. doi: 10.1093/geront/gnaa170, PMID: 33136144 PMC7665461

[45] Hajek A König H-H. Flexible goal adjustment moderates the link between self-rated health and subjective well-being. Findings from the general population. Aging Ment Health. (2021) 25:1345–50. doi: 10.1080/13607863.2020.1765313 32420761

[46] Etxeberria I Etxebarria I Urdaneta E. Subjective well-being among the oldest old: the role of personality traits. Personal Individ Differ. (2019) 146:209–16. doi: 10.1016/j.paid.2018.04.042

[47] Barnay T Juin S. Does home care for dependent elderly people improve their mental health? J Health Econ. (2016) 45:149–60. doi: 10.1016/j.jhealeco.2015.10.008 26608113

[48] Soysal P Veronese N Thompson T Kahl KG Fernandes BS Prina AM. Relationship between depression and frailty in older adults: a systematic review and meta-analysis. Ageing Res Rev. (2017) 36:78–87. doi: 10.1016/j.arr.2017.03.005 28366616

[49] Pearlin LI Bierman A. Current issues and future directions in research into the stress process. In: Aneshensel CS Phelan JC Bierman A, editors. Handbook of the sociology of mental health. Dordrecht: Springer (2013). p. 325–40.

[50] Larsen JT Hershfield H Stastny BJ Hester N. On the relationship between positive and negative affect: their correlation and their co-occurrence. Emotion. (2017) 17:323–36. doi: 10.1037/emo0000231 27709977

[51] Post SG. Altruism, happiness, and health: It’s good to be good. Int J Behav Med. (2005) 12:66–77. doi: 10.1207/s15327558ijbm1202_4, PMID: 15901215

[52] Guo H Sek-yum Ngai S Sun T. Social support and subjective well-being of noncustodial grandparent caregivers in urban China: the mediating roles of generative acts. Geriatr Nurs. (2023) 52:98–105. doi: 10.1016/j.gerinurse.2023.05.010 37290220

[53] Kim KT Hawkins BA Lee Y-H Kim H. Social support and daily life activity: determinants of aging well. Act Adapt Aging. (2023) 47:171–94. doi: 10.1080/01924788.2022.2106013

[54] Zhu C Walsh C Zhou L Zhang X. Latent classification analysis of leisure activities and their impact on ADL, IADL and cognitive ability of older adults based on CLHLS (2008–2018). Int J Environ Res Public Health. (2023) 20:1546. doi: 10.3390/ijerph20021546, PMID: 36674302 PMC9864528

[55] Liu C Luo D Zhou Y Zhang G Feng X Wang Z. Optimism and subjective well-being in nursing home older adults: the mediating roles of gratitude and social support. Geriatr Nurs. (2022) 47:232–8. doi: 10.1016/j.gerinurse.2022.07.020, PMID: 35994812

[56] Chen Y Feeley TH. Social support, social strain, loneliness, and well-being among older adults: an analysis of the health and retirement study. J Soc Pers Relat. (2014) 31:141–61. doi: 10.1177/0265407513488728

[57] Ferguson S Goodwin A. Optimism and well-being in older adults: the mediating role of social support and perceived control. Int J Aging Hum Dev. (2010) 71:43–68. doi: 10.2190/AG.71.1.c, PMID: 20718232

[58] Hobfoll SE. Conservation of resources: a new attempt at conceptualizing stress. Am Psychol. (1989) 44:513–24. doi: 10.1037/0003-066X.44.3.513 2648906

